# Another Tooth-Ache Remedy

**Published:** 1875-07

**Authors:** 


ANOTHER TOOTHACHE REMEDY— Bi-carbonate of soda, a teaspoonful in a tumbler of water and a mouthful of the solution hold in contact with the "raging tooth," will sometimes give immediate relief.

Or, if there is a cavity moisten a little of the soda with a drop or two of laudanum and work the mixture into a paste of proper consistence and then "plug" the tooth.

